# A Rodential Reckoning: A Case Report and Systematic Review of Streptobacillary Endocarditis

**DOI:** 10.4322/acr.2023.423

**Published:** 2023-04-10

**Authors:** Ashwin Mahesh, Eduardo Messias Hirano Padrao, Ravneet Randhawa, Christi Oommen, Johanna Romo, Ramachandra Illindala, Henry Anyimadu

**Affiliations:** 1 University of Connecticut, Department of Medicine, Farmington, CT, USA; 2 Hartford HealthCare, Department of Medicine, Hartford, CT, USA; 3 Hartford HealthCare, Department of Infectious Disease, Hartford, CT, USA

**Keywords:** Rat Bite Fever, Streptobacillus moniliformis, Spirillum minus, Endocarditis

## Abstract

**Introduction:**

Endocarditis is a rare, often fatal complication of rat bite fever caused by *Streptobacillus moniliformis*. Only 39 cases have been reported (including this case) as of 2022. We describe a case and aim to perform this entit’s first systematic literature review.

**Methods:**

We performed a systematic review in CENTRAL, EMBASE, MEDLINE, SciELO, and LILACS. The terms used were terms used were (but not limited to) rat bite fever, *Streptobacillus moniliformis*, *Spirillum minus,* and endocarditis. We included all abstracts and articles with patients with echocardiographic or histologic-proven endocarditis. In case of discordance, a third reviewer was involved. Our protocol was submitted to PROSPERO (CRD42022334092). We also performed searches for studies on the reference list of included articles.

**Results:**

We retrieved 108 and included 36 abstracts and articles. A total of 39 patients (including our report) were identified. The mean age was 41.27, and 61.5% were males. The most common findings were fever, murmur, arthralgias, fatigue, splenomegaly, and rash. Underlying heart disease was present in 33%. Exposure to rats was noted in 71.8% of patients, with 56.4% recalling a rat bite. Anemia was seen in 57%, leukocytosis in 52%, and elevated inflammatory markers in 58% that had lab work performed. The mitral valve was most affected, followed by the aortic, tricuspid, and pulmonary valves. Surgical intervention was required in 14 (36%) cases. Of those, 10 required valve replacement. Death was reported in 36% of cases. Unfortunately, the literature available is limited to case series and reports.

**Conclusion:**

Our review allows clinicians to suspect better, diagnose, and manage Streptobacillary endocarditis.

## INTRODUCTION

Rat bites have historically been associated with febrile disease, with rat bite fever (RBF) being an entity described in medical literature for about 2300 years, first reported in India.^1^ Rodents carry microorganisms in their oral and nasopharyngeal membranes, which can inoculate humans through bites or transdermal contact with urine or feces.^2^

These diseases were initially identified amongst rural communities and populations traditionally exposed to rodents. The advent of pet-keeping and the rise of occupations involving contact with rats, notably laboratory and pet shop workers, in the 21st century led to recent shifts in epidemiological trends in the western world.^3^ The disease has also been noted among snake-keepers, and some authors suggest that rat-consuming snakes could be temporary reservoirs for human infections.^4^ RBF has been attributed to two species of bacteria, *Streptobacillus moniliformis,* and *Spirillum minus*.^1^*Spirillum minus* has been documented in Asian countries, causing the *Sodoku* disease, which roughly translates to “rat-poison”.^2^*Streptobacillus moniliformis* has been documented in North America^5^ causing streptobacillary RBF if transmitted through bites. The disease transmitted via contaminated food or water has been described as Haverhill fever, a name derived from a town in Massachusetts wherein contamination of the raw milk at a local dairy farm gave rise to an epidemic among schoolchildren in 1926.^6^*Streptobacillus moniliformis* is a non-motile, microaerophilic, Gram-negative rod-shaped bacterium that is a member of the family *Leptotrichiaceae*. It exhibits slow growth on anaerobic blood cultures, making laboratory identification challenging.^3,7^

RBF is a rare disease with a mortality rate of 13%.^1^ The mortality rate is higher with complications like septic shock and endocarditis. The data on endocarditis is limited owing to the rarity of the disease, possible underdiagnosis, and underreporting. To our knowledge, only 38 cases have been reported between 1915 and 2022. We describe a patient who presented with an acute stroke weeks after she said being bitten by a rat and was later found to have infective endocarditis of her native mitral valve with cultures yielding *Streptobacillus moniliformis.* We also present a systematic review of all the cases of *Streptobacillus moniliformis* endocarditis reported to date.

## CASE REPORT

A 75-year-old healthy, independent Caucasian female presented from home, endorsing worsening back pain for a week that limited her ability to walk around the house. She mentioned being bitten by a pet rat a few weeks prior, with subjective fevers and chills lasting for a few days after the bite without accompanying rashes or skin changes. She denied a personal history of intravenous drug use. She did not have previous valvular surgeries or any previous valvular lesions. Her medical history included osteoarthritis and hypertension.

At the emergency room, vitals were significant for a temperature of 37.6C, and pulse was measured at 86 beats per minute, respirations at 18 breaths per minute, blood pressure at 110/64 mm Hg with 97% oxygen saturation on room air. Her physical exam was significant for a systolic 2+ high-pitched murmur on the left fifth intercostal space on the midclavicular line. Her oral exam revealed poor dentition with caries, and the site of her reported rat bite on her right index finger showed a small well-healed wound. Labs on admission revealed a white blood cell count of 11.8x10^3^/mm^3^ (reference range [RR]: 4-11.8x10^3^/mm^3^), hemoglobin 10.1 g/dL (RR: 13-17.7g/dL), platelets 220x10^3^/mm^3^ (RR: 150-450x10^3^/mm^3^), sodium 138 mEq/L (RR: 136-145 mEq/L), potassium 3.5 mEq/L (RR: 3.4-5.3 mEq/L), blood urea nitrogen 15 mg/dL (RR: 8-21mg/dL), creatinine 0.5 mg/dL (RR: 0.5-1.3 mEq/L). High-sensitivity troponins were initially measured at 59.22 ng/L (RR: <14ng/L), with a repeat drawn a few hours later measured at 57.78 ng/L. An electrocardiogram on admission revealed sinus rhythm with no concerning ST-T wave changes with a few premature atrial contractions at a rate of 98 beats per minute. A chest X-ray revealed bibasilar opacities concerning atelectasis.

Cardiology was consulted for the troponin leak and recommended a transthoracic echocardiogram that revealed a preserved ejection fraction with no regional wall abnormalities. Valvular vegetations were initially not discerned. Given the absence of clinical features of the acute coronary syndrome, conservative management was pursued with plans for an outpatient ischemic evaluation.

**Figure 1:**
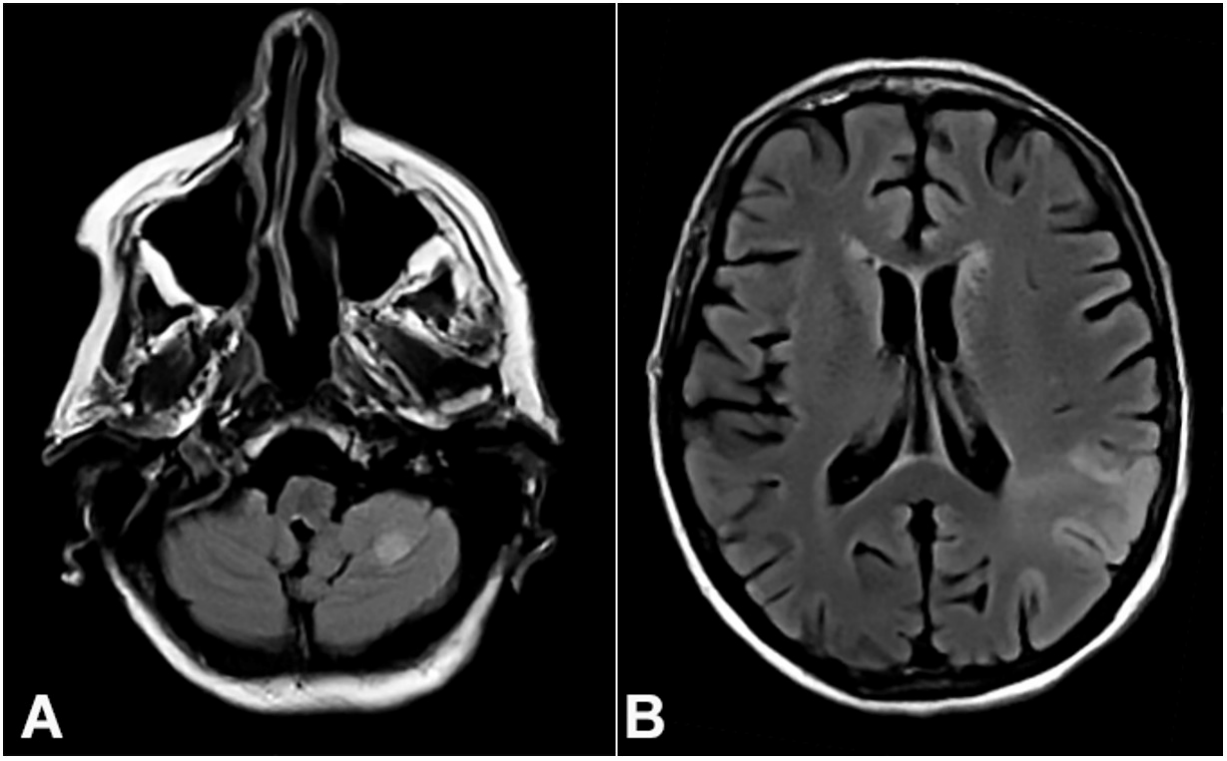
FLAIR MRI section showing in **A -** strokes in the left cerebellar; and in **B -** left parietal (B) regions of the brain, concerning an embolic etiology. (FLAIR: Fluid Attenuated Inverse Recovery; MRI: Magnetic Resonance Imaging)

**Figure 2:**
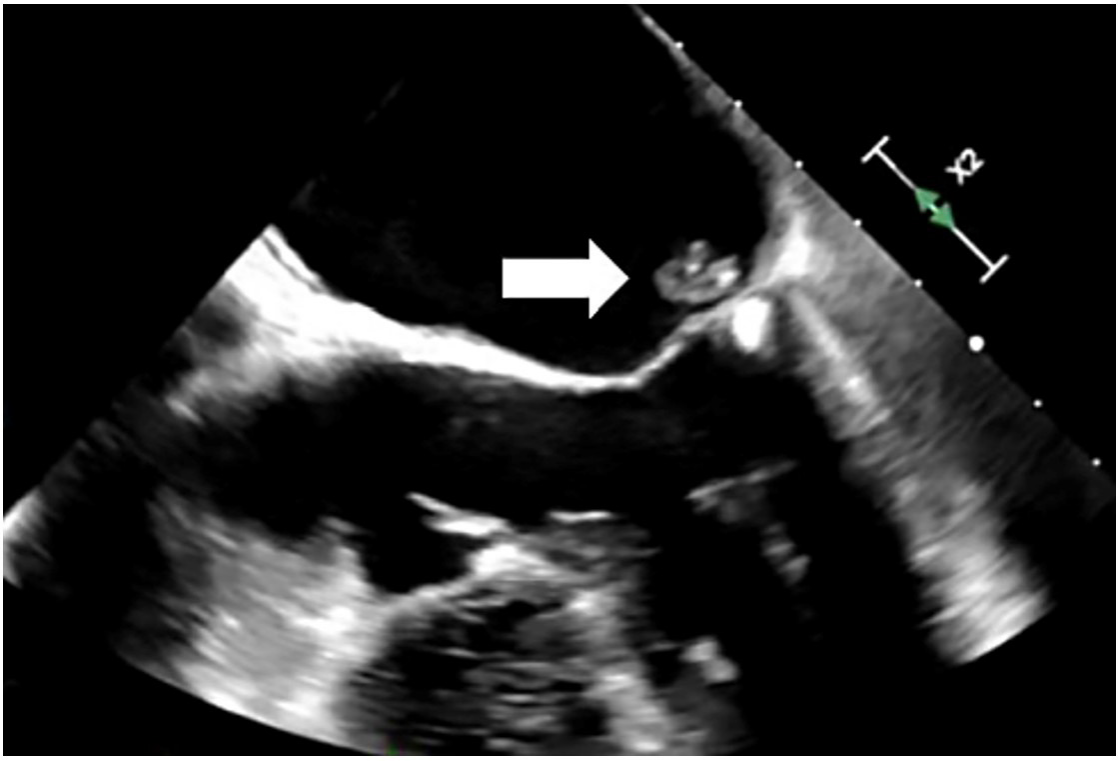
Trans-esophageal echocardiography showing a 12 mm-sized mobile atrial vegetation with marked thickening of the posterior mitral leaflet.

On day three of hospitalization, she underwent a spine MRI that revealed a herniated disc at T11/T12. Following the scan, she experienced a new onset of verbal aphasia. CT imaging and angiography of the head and neck were unremarkable. She spiked a fever of 39.3C later that day. After repeat blood cultures, she was started on ceftriaxone and azithromycin for suspicion of lobar pneumonia based on new infiltrates on a repeat chest x-ray. Given persistent aphasia, she underwent a brain MRI that showed acute infarcts in the left parietal and frontal lobes and cerebellum (Figure 1), consistent with an embolic etiology. A transesophageal echocardiogram revealed marked thickening of the posterior mitral leaflet with a 12 mm complex echodensity adherent to the atrial surface (Figure 2). This was accompanied by moderate eccentric and posteriorly directed mitral regurgitation.

Blood cultures later revealed Gram-negative rods on the Gram stain in one of four bottles, speciated as *Streptobacillus moniliformis*. A peripherally inserted central line was subsequently placed, and she was prescribed a 6-week course of IV ceftriaxone at 1g/day. Her aphasia improved, and she was safely discharged with outpatient follow-up with Infectious disease.

## METHODS

Two independent reviewers (AM and EMHP) performed a search in CENTRAL (Cochrane database), EMBASE, MEDLINE (PubMed), SciELO (Scientific Electronic Library Online), and LILACS (Latin American and Caribbean Health Sciences Literature). Terms used were, but not limited to “rat bite fever” (“rat-bite”, “rat bite”, “rat-bite fever”[Mesh], “ratbite”, “*Sodoku*”, “Haverhill”), *Streptobacillus moniliformis*, *Spirillum minus*, and endocarditis (“endocarditis” and “endocarditides”). Further details regarding the keywords have been provided in the Supplement Appendix.

The search was performed between April 28th to 30th, 2022. We included all abstracts and articles describing characteristics of patients with echocardiographic or histologic-proven (biopsy or autopsy) endocarditis due to *Streptobacillus moniliformis* or *Spirillum minus*. The pediatric and adult populations were included. Duplicated retrievals were excluded. In case of discordance, a third reviewer (CO) was involved in a final decision. We excluded articles, not in English, Portuguese, French, or Spanish. We also searched for studies on the reference list of the included articles.

The information was extracted by AM and EMHP. It included sex, age, symptoms, laboratory findings, diagnosis of endocarditis, echocardiogram findings, involved valves, vegetation size, treatment and surgery performed, complications, and outcomes. Since this was a qualitative and systematic review, we did not perform a meta-analysis. Our protocol was submitted to PROSPERO (CRD42022334092).

## RESULTS

**Figure 3:**
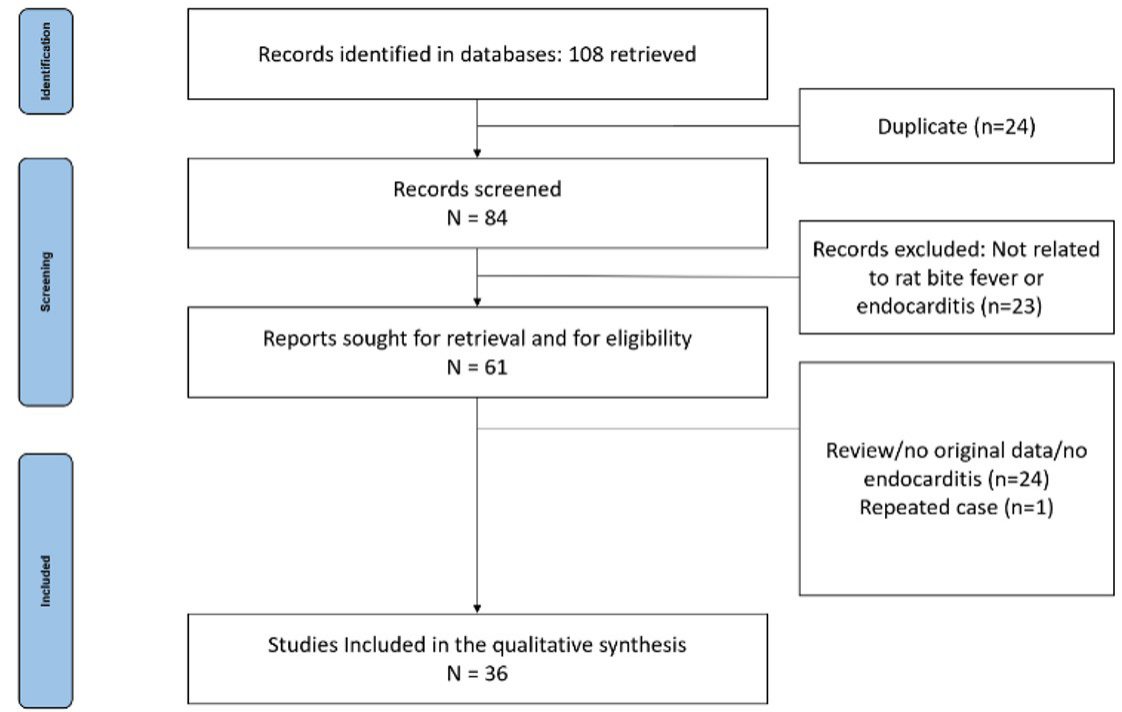
Flow diagram.

We retrieved 108 results, and we included 36 abstracts and articles. Figure 3 shows the flow diagram according to PRISMA guidelines.

There were a total of 39 cases reported^7-41^ (including our case). We excluded one duplicated case^42^. Table 1 presents the initial findings and work-up of rat bite endocarditis, while Table 2 presents the complications, treatment, and outcomes.

**Table 1:** Initial findings and work-up of rat bite endocarditis

***Year***	***Age, Sex***	***Heart Disease***	***Rat Exposure***	***Presentation***	***Exam***	***Work-up***	***Echo***	***Blood Cultures***
1915^8^	67; F	None	Rat Bite	Fever	Fever, rash, murmur	NR	None	None
1934^9^	18; M	RHD	NR	NR	NR	NR	None	None
1940^10^	14; F	None	Rat Bite	Fever	Fever, murmur	Anemia	None	None
1944^11^	43; M	RHD	Rat exposure without known bite	Fever, rash	Fever, rash, murmur	NR	None	None
1945^12^	22; M	RHD	NR	NR	Murmur, petechiae	NR	None	None
1947^13^	17; F	NR	NR	NR	NR	NR	None	None
1949^12^	40; M	RHD	Rat exposure without known bite	Fever, fatigue, weight loss	Murmur, splenomegaly, Osler nodes, water-hammer pulse, Duroziez sign	Hg 11 g/dL, WBC 6500 x10^8^ cells/L (PMN predominance), ESR 13 mm/hr, EKG with PVCs, LVH	None	None
1949^14^	27; M	RHD	Handled a dead rat	Fever, anemia	Fever, murmur, splenomegaly, Osler nodes	Anemia	None	None
1952^15^	54; M	None	Rat Bite	Fever, rash, headache, arthralgia, chest discomfort	Fever, murmur, Osler nodes, nausea, documented rat bite	WBC 17500 x10^8^ cells/L, Hg 13.5 g/dL (PMN predominance)	None	None
1967^16^	70; F	Calcific Aortic Stenosis	Unknown	Fever, nausea and weight loss	Murmur, splenomegaly	Hg 8.8 g/dL, WBC 12300 x10^8^ cells/L (PMN predominance)	None	None
1967^16^	43; M	NR	Rat Bite	Fever, arthritis, with pleuritic chest pain, heart failure	Fever, murmur, cardiac tamponade (atrial fibrillation on EKG)	Hg 13.8 g/dL, WBC 16500 x109 cells/L,	None	NR
1967^17^	60; M	RHD	Rat exposure without known bite	Fever, tonic-clonic generalized seizures, weight loss, altered mental status	Fever, murmur, clubbing	Hg 13.5 g/dL, WBC 14200 x10^8^ cells/L (PMN predominance), CSF with 521 cells.	None	None
1974^18^	55; F	NR	NR	Anorexia, weakness and myalgia	NR	WBC 17,900 x10^8^ cells/L with 76% PMN.	None	Positive
1981^19^	41; M	None	Rat Bite	Fevers, murmur, and heart failure	NR	NR	None	None
1985^20^	3m; M	None	Rat Bite	Fevers, lethargy, and arthritis	Fever, hepatosplenomegaly, and indentation at site of reported rat bite	Hg 11.3 g/dL, WBC 9800 x10^8^ cells/L, interstitial pneumonia on chest imaging	None	None
1985^20^	63; F	None	Rat Bite	Fever, arthritis, paresis	Fever, murmur	NR	None	None
1986^21^	8; M	RHD	None	Fever, lethargy, weight loss	Murmur, hepatosplenomegaly, clubbing	WBC 12,500 x10^8^ cells/L (PMN predominance) first degree AV block	Aortic stenosis with regurgitation without vegetations	None
1989^22^	2m; M	None	Rat Bite	Fever, pneumonia, “symptoms concerning for meningitis”	Hepatosplenomegaly	Electrolytes WNL on basic chemistry panel	None	None
1992^23^	46; M	None	Rat Bite	Fevers, back pain, arthralgia/arthritis	Fever, murmur, hepatosplenomegaly	Hg 10g/dL, WBC 8200 x10^8^ cells/L (PMN predominance), ventricular bigeminism	Thickened aortic valve	Positive
2000^24^	37; M	None	Rat Bite	Arthralgia, fever and history of HIV	Fever, pustules on skin	Hg 9.3g/dL, WBC 6700 x10^8^ cells/L, PMN predominance, platelets 195 x10^8^ cells/L	Vegetation on mitral valve with rupture	Positive
2004^25^	24; M	None	Patient reportedly scratched finger on a rat cage	Fever, chills, myalgia, arthralgia, shortness of breath, vomiting, wound	Wound on physical exam, murmur, and pericardial effusion	WBC 16100 x10^8^ cells/L, PMN predominance	Aortic Valve Regurgitation	Positive
2006^26^	18; M	Small VSD	Rat Bite	Fevers, cough, epistaxis, palpitations, and arthralgia	Fever	WBC 7,600 x10^8^ cells/L, platelets 127,000 x10^8^ cells/L, ESR 70 mm/hr	Tricuspid Valve vegetation with small VSD	Positive
2007^27^	29; M	None	Rat Bite	Fevers, dyspnea, dizziness, and somnolence	Fever	NR	Large vegetations on the aortic valve. Severe Aortic regurgitation and LV dysfunction	Negative
2007^28^	74; F	None	NR	Fever and murmur	NR	NR	Severe Mitral Valve regurgitation with vegetation	Negative
								Positive mitral valve culture
2007^29^	60; F	Mechanical Mitral Valve	Rat Bite	Fever, leg wounds, weakness, and weight loss	NR	WBC 15,100 x109 cells/L, PMN predominance; Hg 8.2 g/dl; Platelets 134,000 x109 cells/L. CRP 162.9 mg/l, ESR 14 mm/h	Mechanical mitral valve dehiscence with severe mitral regurgitation and vegetations	Negative
2010^30^	35; M	Tetralogy of Fallot	Rat Bite	Vomiting and diarrhea followed by the development of debilitating migratory, asymmetric polyarthralgia, irregularly relapsing fevers	Rash involving palms and soles		A 2 cm vegetation on tricuspid valve and a 0.5 cm vegetation on the aortic valve	Negative
2011^31^	45; M	None	No	Rash and right ankle pain	Erythema	WBC 28,760 x109 cells/L, PMN predominance	Vegetation on mitral valve	Negative
2013^32^	44; M	None	Rat Bite	Fever	NR	NR	Mitral valve vegetation with severe regurgitation	Positive
2014^33^	49; M	None	None	Fever, rash, leg swelling	Pansystolic murmur, splinter hemorrhage	Hb 10.5 g/dL, WBC WNL, CRP 117 mg/dL, HIV serology negative	Mitral valve vegetation and thickening	Positive
2017^34^	19; M	Tetralogy of Fallot	Rat Bite	Fever, chest pain, myalgias, weight loss	NR	NR	Large fistula between right sinus of Valsalva and RV. Vegetations on VSD and tricuspid valve	Negative blood culture, operative cultures positive
2018^35^	52; F	None	Rat bite	Lethargy, fever, sepsis, right knee and ankle arthritis	Punctate opening in the heel of right foot	WBC 22,600 x109 cells/L with PMN predominance, ESR 85 mm/hr, CRP >270 mg/dL	Mitral valve vegetation with perforation and regurgitation	Negative blood cultures, positive synovial fluid cultures, mitral valve cultures
2018^36^	33; F	None	Rat Exposure	Fever, arthralgia, myalgia, flu-like symptoms, dyspnea, murmur	NR	WBC 11,700, PMN predominance	Left atrial mass with severe mitral regurgitation	Positive
2018^37^	7m; M	Tetralogy of Fallot	Rat bite	Fever, hypoxia	NR	The initial blood work WNL, but mild elevation of CRP.	Pulmonary valve vegetation, destruction of native pulmonary valve and pulmonary artery with aneurysm	Negative
2019^38^	47; M	None	None	Pain and fever	NR	WBC elevation, inflammatory marker elevation	Mitral and aortic insufficiency and vegetation	Negative
2019^39^	24; F	None	NR	Bilateral lower extremity hemiparesis, bowel and urinary incontinence, weight loss, fever, dysarthria, limb ischemia	Decubitus ulcer	NR	A 4.5 cm mitral vegetation	Negative
2020^40^	65; M	None	Rat bite	Fevers, Myalgias, splenic abscess	NR	CRP 135 mg/L, Hb 7.3 g/dL, WBC 6400, Platelets 167,000 EKG with sinus rhythm and RBBB	Severe mitral and Aortic vegetations with aortic root abscess	Positive
2020^7^	24; F	None	Rat Bite	Pregnant women	Murmur, splenomegaly	Anemia, WBC WNL, arterial popliteal thrombosis, renal and splenic infarcts on imaging	2 cm mitral valve vegetation	Negative
*2021^Our case^*	75; F	None	Rat bite	Stroke, fever	Murmur, rat bite on finger	Hg 10.1 g/dL WBC 11.8 x10^8^ cells/L, EKG with PACs	12 mm Mitral vegetation moderate mitral regurgitation	Positive
2022^41^	44; M	None	Rat Bite	Arthralgias, lethargy, fevers, and rigors	Fever	NR	13 mm mitral valve mobile mass, mitral regurgitation	Positive

AV - atrioventricular; CRP - C-reactive protein; CSF - cerebral spinal fluid; EKG - electrocardiogram; ESR - erythrocyte sedimentation rate; Hg - hemoglobin; HIV - Human immunodeficiency virus infection; LV - left ventricle; LVH - left ventricle hypertrophy; m - month; NR - not reported; PAC - premature atrial contraction; PMN - polymorphonuclear; PVC - premature ventricular complex; RBBB - right bundle branch block; RHD - rheumatic heart disease; TOF - tetralogy of fallot; VSD - ventricular septal defect; WBC - white blood cell; WNL- within normal limits.

**Table 2:** Complications, treatments offered and outcomes

***Year***	***Age, Sex***	***Antibiotic Treatment***	***Surgery***	***Outcome***
1915^8^	67; F	None	None	Died
1934^9^	18; M	None	None	Died
1940^10^	14; F	None	None	Died
1944^11^	43; M	Penicillin	None	Died
1945^12^	22; M	Penicillin	None	Died
1947^13^	17; F	Penicillin	None	Died
1949^12^	40; M	Penicillin	None	Cured
1949^14^	27; M	Penicillin and Chloramphenicol	None	Cured
1952^15^	54; M	Penicillin and Streptomycin for 3 weeks	None	Cured
1967^16^	70; F	Penicillin	None	Cured
1967^16^	43; M	Penicillin	None	Died
1967^17^	60; M	Penicillin	None	Cured
1974^18^	55; F	Cephalothin and gentamicin	None	Died
1981^19^	41; M	Penicillin, gentamycin	None	Sudden death 4 months after treatment
1985^20^	3m; M	No treatment	None	Died
1985^20^	63; F	Penicillin, amikacin	None	Cured
1986^21^	8; M	Penicillin per day	None	Cured, but died 2 months later due to rheumatic heart disease
1989^22^	2m; M	None	None	Died
1992^23^	46; M	Penicillin, tetracycline	None	Cured
2000^24^	37; M	Ceftriaxone for 3 weeks, gentamycin for 2 weeks and penicillin for 1 week	None	Cured
2004^25^	24; M	Ceftriaxone	Debridement, aortic valve replacement	Died
			Second approach with new debridement and mitral valve repair	
2006^26^	18; M	Penicillin, gentamycin for 4 weeks	None	Cured
2007^27^	29; M	Ampicillin, ceftriaxone, gentamicin	Aortic Valve Replacement	Cured
		Post-operative Fosfomycin and gentamycin		
2007^28^	74; F	Penicillin G for 4 weeks	Mitral valve replacement	Cured
2007^29^	60; F	Levofloxacin daily, ceftriaxone, gentamycin, oral doxycycline	Mitral valve replacement	Cured
2010^30^	35; M	Ampicillin, sulbactam and gentamicin	None	Cured
2011^31^	45; M	Cloxacillin, ampicillin and gentamicin, ampicillin for 5 days followed by piperacillin-tazobactam and vancomycin completing for 10 days.	Mechanical mitral valve repair	Cured
2013^32^	44; M	Doxycycline oral for two days, amoxicillin for one day, penicillin for 6 weeks with for 2 weeks	None	Cured
2014^33^	49; M	Penicillin and oral doxycycline	Bioprosthetic mitral valve replacement	Cured
2017^34^	19; M	6 weeks of penicillin and two weeks of gentamicin	Surgical repair, debridement, aortic mechanical valve replacement and bioprosthetic pulmonary valve replacement	Cured
2018^35^	52; F	Vancomycin and piperacillin-tazobactam initially for septic arthritis, ceftriaxone for 6 weeks after	Mitral valve repair	Cured
2018^36^	33; F	Ceftriaxone	Mitral valve repair	Cured
2018^37^	7m; M	Ceftriaxone for 6 weeks	None	Cured
2019^38^	47; M	Piperacillin Tazobactam, azithromycin, clotrimazole followed by ceftriaxone and ampicillin with daptomycin.	Mitral and Aortic Valve replacement	Cured
2019^39^	24; F	Not mentioned antibiotic course, but received antibiotics	Mitral valve replacement	Cured
2020^40^	65; M	Cefuroxime, later switched to meropenem	Bioprosthetic valve replacement of aortic, mitral valves, debridement of aortic root abscess	Died
2020^6^	24; F	Penicillin for 6 weeks, amoxicillin-clavulanate for 2 weeks	Mitral valve repair with band	Cured
*2021^Our case^*	75; F	Ceftriaxone for 6 weeks	None	Cured
2022^41^	44; M	NR	Vegetectomy and mitral valve repair with bovine patch	Cured

NR - not reported.

The mean age was 41±17 years, and 61.5% of the patients were males. Four of them were younger than 18-year-old. Underlying heart diseases accounted for 13 cases (33%), and rheumatic heart disease was the most common (20.5%). Rat exposure history was present in 71.8%; however, only 56.4% of the patients were able to profess a history of a bite. The rat bite indentation was only seen in 3 cases (7.7%). Most common symptoms included fever (84.6%), murmur (54%), arthralgia/arthritis (31%), fatigue (31%), splenomegaly (20.5%), and skin findings (25.6%). When available, anemia was seen in 57%, leukocytosis in 52%, and elevated inflammatory markers were seen in 58%. The most common valve affected was the mitral valve (63.6%), followed by the aortic (31.8%), tricuspid (9%), and pulmonary (4.5%) valves. Embolization was reported in 6 cases. The most commonly used antibiotic was penicillin (48.7%), followed by ceftriaxone (23%). Other antibiotics include chloramphenicol, streptomycin, cephalothin, gentamicin, amikacin, tetracycline, doxycycline, piperacillin-tazobactam, ampicillin, levofloxacin, and others. In our review, surgical intervention was required in 14 (36%) cases; of those, 10 required valve replacement. Death was reported in 36% of cases. Table 3 summarizes the most common presentations, laboratory work-up, complications, echocardiogram findings, and mortality.

**Table 3:** Summary of findings

**Characteristic**	**Frequency**
**Age (Mean - SD years)**	41.27±17.4 years
**Male Sex**	24/39 (61.5%)
**Rat Exposure**	28/39 (71.8%)
**Rat Bite**	22/39 (56.4%)
**Underlying Heart Disease**	13/39 (33.3%)
Rheumatic Heart Disease	7/39 (17.9%)
Tetralogy of Fallot	3/39 (7.6%)
Ventricular Septal Defect	1/39 (2.5%)
Mechanical Valve	1/39 (2.5%)
Calcific Aortic Stenosis	1/39 (2.5%)
**Signs and Symptoms**	
Fever	33/39 (84.6%)
Weight Loss	7/39 (17.9%)
Fatigue	12/39 (30.8%)
Murmur	21/39 (53.8%)
Myalgia	6/39 (15.4%)
Arthralgia/Arthritis	12/39 (30.8%)
Hepatomegaly	5/39 (12.8%)
Splenomegaly	8/39 (20.5%)
Rat Bite Punctate Lesion	3/39 (7.7%)
Skin Findings	10/39 (25.6%)
**Laboratory Work-up**	
Anemia - Hemoglobin (Mean - SD)	12/21 (57.1%) 10.6±2.1g/dL
Leukocytosis - Mean WBC (Mean - SD)	11/23 (52.2%) 13550±5943/mm^3^
Inflammatory Markers Elevation^*^	7/12 (58.3%)
**Embolization**	6/23 (17.1%)
**Echocardiogram Findings** *Valve Affected*	
Aortic Valve	7/22 (31.8%)
Mitral Valve	14/22 (63.6%)
Tricuspid Valve	2/22(9.1%)
Pulmonary Valve	1/22 (4.5%)
**Mortality**	14/39 (35.9%)

SD - standard deviation.

* Either CRP or ESR elevation.

## DISCUSSION

The clinical features of RBF encompass a variety of non-specific symptoms, challenging the diagnosis. Notable symptoms documented were fevers in 30% of patients, arthralgias and arthritis in 49%, lymphadenopathy in 25%, and morbilliform or petechial rash in 75%.^1,2^ The onset of symptoms is usually as early as 3 days and up to 2-3 weeks after exposure.^1,2^ Endocarditis is a rare complication of RBF, and only 39 cases have been reported.^7-42^ Per our review, the mean age is 41 years, males are more affected, and children are affected in 50% of patients. As opposed to uncomplicated rat bite fever, streptobacillary endocarditis seems to have more specific symptoms. Rat bite fever usually manifests with fever, migratory polyarthralgia, and a rash, maculopapular or purpuric. Most patients with rat bite endocarditis present with a murmur, and almost one-fourth present with splenomegaly.^2^ Arthralgia, arthritis, and skin lesions are also common in rat bite endocarditis. The skin rash may also show findings typical of endocarditis, such as Osler nodes and splinter hemorrhages. As with any endocarditis,^43^ underlying heart disease seems to be a risk factor. Interestingly there were 2 case reports of *Streptobacillus moniliformis* leading to endocarditis in patients with Tetralogy of Fallot,^30,37^ and only one case in a patient with a mechanical valve.^29^

Unfortunately, concerning laboratory workup, almost one-third of the cases were reported in the first half of the 20th century [Cases Reported prior to1950], and many did not provide any laboratory workup. Anemia, leukocytosis, and elevated inflammatory markers are common findings.

The mitral valve is the most commonly affected valve accounting for 63.6% of the cases, followed by the aortic (31.8%), tricuspid (9%), and pulmonary (4.5%) valves. A similar pattern of valve involvement is seen in other infective endocarditis. Embolization does not seem to be a common phenomenon since there are only 4 cases, although the overall incidence of embolization in infective endocarditis, it is up to 44%.^44^ Organs affected in our series included the spleen, kidney, and brain.

Given the limited number of cases, there was variability in antibiotic choices for the treatment. The most commonly used antibiotic was penicillin. Ceftriaxone was the second most used antibiotic. Other antibiotics include chloramphenicol, streptomycin, gentamicin, amikacin, tetracycline, doxycycline, amoxicillin, piperacillin-tazobactam, cefuroxime, and meropenem. For our patient, we used ceftriaxone since it is considered one of the first-line antibiotics for rat bite fever with no endocarditis^1^ and has been extensively and successfully studied for other etiologies of endocarditis.^43,45^ The role of surgical intervention is based on factors like abscess formation >1cm, poor response to antibiotics, large vegetations with embolic events, and heart failure or the development of cardiogenic shock.^43,45^ In our review, surgical intervention was required in 14 cases. Of those, 10 required valve replacement.

Death has been reported in 13 cases (36%); however, 6 of them occurred in the first half of the 20th century, and only 7 of the deaths were patients who failed antibiotic therapy. The mortality of rat bite endocarditis is much higher when compared to uncomplicated rat bite fever, which, when untreated, can cause death in up to 13% of patients.^1^

## CONCLUSION

This case comprises an interesting series of events that began with a rat bite and eventually culminated with the identification of vegetation on the valves of the heart and *Streptobacillus moniliformis* in the blood. Our systematic review aims to shed light on a rare complication of a disease with high morbidity and mortality. Understanding clinical profiles may help clinicians better suspect, diagnose, and manage Streptobacillary endocarditis.

## Supplementary Material

Supplementary material accompanies this paper.

Supplement Appendix - Keywords

This material is available as part of the online article from https://doi.org/10.4322/acr.2023.423
