# Supplementary material for: A Rodential Reckoning: A Case Report and Systematic Review of Streptobacillary Endocarditis
Source: Autops Case Rep. 2023 Apr 10;13:e2023423. doi: 10.4322/acr.2023.423 (PMC10124564; doi:10.4322/acr.2023.423)
Supplement: Supplement Appendix - Keywords [file autopsy-13-e2023423-Supl.pdf]

Supplement Appendix - Keywords

**Keyword**	**Text Word**	**Mesh Terms**
Rat-bite Fever	“Rat-bite” OR “Rat bite” OR “Ratbite” OR “Spirillum minus” OR “Haverhill” OR “Streptobacillus moniliformis” OR “Sodoku”	"Rat-Bite Fever"[Mesh]
Endocarditis	“Endocarditis” OR “Endocarditides”	"Endocarditis”[Mesh]
